# Plasma and Urinary Neutrophil Gelatinase-Associated Lipocalin as Predictors of Renal Parenchymal Involvement in Children with Febrile Urinary Tract Infection: A Pilot Study

**DOI:** 10.3390/children11091081

**Published:** 2024-09-03

**Authors:** Emma Baranton, Chloé Ribet, Emma Freyssinet, Julie Bernardor, Corinne Boyer, Florence Lavrut-Hollecker, Diane Demonchy, Emma Schuler, Eric Fontas, Antoine Tran

**Affiliations:** 1 Pediatric Emergency Department, Lenval Children’s Hospital, 06200 Nice, France; emma.baranton@etu.univ-cotedazur.fr (E.B.);; 2 Pediatric Nephrology Department, University Hospital of Nice Archet 2, 06200 Nice, France; 3 Medical School, Université Côte d’Azur, 06107 Nice, France; 4 Department of Radiology, Lenval Children’s Hospital, 06200 Nice, France; 5 Department of Polyvalent Biology, University Hospital of Nice Pasteur, 06002 Nice, France; lavrut-hollecker.f@chu-nice.fr; 6 Department of Clinical Research, University Hospital of Nice Cimiez, 06003 Nice, France; fontas.e@chu-nice.fr

**Keywords:** urinary tract infection, acute pyelonephritis, neutrophil gelatinase-associated lipocalin, NGAL, children, MRI

## Abstract

Background: Urinary tract infections (UTIs) are very common bacterial infections in children. Early detection of renal parenchymal involvement in this setting can help clinicians make more effective treatment choices. The aim of this pilot study was to assess the ability of plasma and urinary neutrophil gelatinase-associated lipocalin (pNGAL and uNGAL) levels, measured using an automated system, to accurately predict renal parenchymal involvement in children with febrile UTIs. Methods: This prospective single-center study included 28 children aged ≥ 4 years with a first episode of febrile UTIs. All patients underwent magnetic resonance imaging. pNGAL, uNGAL, procalcitonin, C-reactive protein (CRP), and white blood cells were measured before antibiotic therapy. Results: The receiver operating characteristic (ROC) area under the curve for predicting acute pyelonephritis was 0.6 for pNGAL, 0.8 for CRP, 0.4 for PCT, and 0.4 for uNGAL. The ROC analyses showed an optimal cutoff of 141.0 ng/mL for pNGAL (sensitivity, 54.2%; specificity, 75.0%; positive predictive value, 92.9%; and negative predictive value, 21.4%). Conclusion: pNGAL and uNGAL did not effectively aid the early prediction of renal parenchymal involvement in children ≥ 4 years with febrile UTIs. The novelties of this study were the use of MRI as the gold standard and an automated biochemical method to measure NGAL.

## 1. Introduction

Urinary tract infections (UTIs) are very common in infants [1,2]. Differentiating between lower and upper infections based on clinical symptoms alone is challenging, particularly in young children, who often have nonspecific symptoms [3].

Although DMSA scans are the gold standard for detecting renal parenchymal involvement [4], they are not typically used during the acute phase of infection, as they are invasive, costly, time-consuming, not widely accessible, and involve exposure to radiation [5]. Urine culture is the test of choice for diagnosing UTIs in routine practice, but results normally take about 2–3 days to come back [6].

A prompt and accurate diagnosis of parenchymal involvement is important in UTIs, as delayed initiation of antibiotic therapy can cause short-term complications, such as bacteremia and severe sepsis, and long-term complications, such as renal scarring [7,8], high blood pressure, and even chronic kidney failure [9]. Early scintigraphy detects renal parenchymal involvement in just 50–64% of children with febrile UTIs [10]. As this test is not routinely performed [6], all febrile UTIs are considered to be acute pyelonephritis (APN) and are treated as such. According to the American Academy of Pediatrics guidelines, oral and parenteral antibiotics are equally effective for the treatment of febrile UTIs. European guidelines, however, recommend basing the choice of administration route on pharmacokinetic and pharmacodynamic parameters and the emergence of ESBL-producing *Escherichia coli* [11]. Oral antimicrobial therapy is the recommended treatment for uncomplicated, nonsevere UTIs, defined by clinical parameters (age > 3 months, absence of underlying uropathy, sepsis, and vomiting, low procalcitonin (PCT) levels, good compliance, and the possibility of reconsulting where necessary), as it is assumed that these patients do not have renal impairment. Overdiagnosis of APN is also problematic, as it leads to overuse of broad-spectrum antibiotics, favoring the emergence of resistant bacteria. Appropriate urine collection can reduce false positives, but again, it is challenging in children.

Considering the above, more practical and rapid tools would be useful for guiding treatment strategies [12]. Traditional markers of infection, such as serum C-reactive protein (CRP), PCT, and white blood cells (WBCs), are a useful adjunct for diagnosing APN in children, but their performance in this setting is limited.

Based on animal models and studies in humans, neutrophil gelatinase-associated lipocalin (NGAL), a glycoprotein in the lipocalin superfamily, is an effective marker of acute kidney injury [13,14,15]. Plasma NGAL (pNGAL) is secreted by neutrophils in response to systemic inflammation, while urinary NGAL (uNGAL) is secreted by alpha-intercalated cells following damage to the genitourinary epithelium [16]. These cells are found in the renal collecting duct.

A number of recent studies have shown that NGAL may be useful for predicting APN [17,18,19,20,21], but contradictory results have also been reported [22,23,24]. To our knowledge, positivity thresholds for pNGAL and uNGAL vary according to the cohort, assay method, and gold standard used. These past years, NGAL was difficult to use in routine practice, as levels were typically measured using nonautomated methods. Many clinicians now have access to a new automated turbidimetric immunoassay method, which could be used to automate the quantification of NGAL in plasma and urine samples.

The aim of this pilot study was to assess the ability of pNGAL and uNGAL levels measured using an automated method to predict renal parenchymal involvement in children with febrile UTIs, with confirmation by renal diffusion-weighted MRI.

## 2. Materials and Methods

### 2.1. Patient Characteristics and Inclusion Criteria

This was a prospective single-center study conducted at the Lenval Children’s Hospital in Nice, France, between March 2020 and April 2023. The original protocol contemplated a multicenter study comprising 2 additional hospitals, in Antibes and Grasse, but because MRI was not available at these hospitals, they were finally not included.

Candidates for inclusion were children aged ≥ 4 years with their first febrile UTIs, defined as fever (body temperature ≥ 38.5 °C) and a positive urine culture (pyuria ≥ 10^4^ WBCs/mL and monomicrobial bacteriuria culture ≥ 10^5^ organisms/mL). Children with known urinary tract abnormalities or malformations, a past history of UTIs, current antibiotic therapy, or antibiotic prophylaxis 48 h before admission were excluded. Following inclusion, the children were divided into two groups—an APN group and a lower UTI group—depending on whether or not renal cortical defects were observed by magnetic resonance imaging (MRI). Urine samples for culture were collected via catheterization or midstream catch with local disinfection.

This study was conducted in accordance with the principles of the Declaration of Helsinki and the protocol was approved by the local ethics committee (project identification code no. 19.07.15.46852 with RCB ID: 2019-A01942-55). Written informed consent was obtained in advance from all the children’s parents.

### 2.2. Laboratory and Imaging Studies

CRP, PCT, WBCs, and neutrophils were measured on admission, before the administration of antibiotics. NGAL samples were collected in the emergency department, also before initiation of antibiotics, and frozen. At the end of the study, they were measured using the Bioporto automated turbidimetric immunoassay (Roche) (detection range, 50–5000 ng/mL). MRI was chosen as the reference standard due to its minimally invasive nature (greater likelihood of parents agreeing to their children participating in the study) and because recent studies have shown it to be equal or even superior to DMSA [25,26,27,28] for detecting renal parenchymal involvement. The MRIs were obtained in the first week of admission and interpreted by two pediatric radiologists at the Lenval Children’s Hospital. Renal parenchymal involvement (foci of nephritis) was diagnosed when diffusion sequences showed high signal intensity areas and apparent diffusion coefficient maps showed low signal intensity areas. Laboratory, clinical, and imaging results were compared between children with APN and those with lower UTIs.

### 2.3. Statistical Analyses

The results were summarized using standard descriptive statistics. Groups were compared using the unpaired *t*-test for quantitative data and the chi-square test for qualitative data. The diagnostic performance of the biomarkers—CRP, PCT, WBCs, neutrophils, pNGAL, and uNGAL—was investigated using receiver operating characteristic (ROC) curves, with the calculation of optimal cutoffs for predicting APN. The area under the curve (AUC) for each biomarker was calculated. Correlation graphs between biomarkers were generated, with the calculation of Pearson’s correlation coefficients. A *p*-value < 0.05 was considered statistically significant. Statistical analyses were performed in SAS Enterprise Guide v7.1 (SAS Institute Inc, Cary, NC, USA).

## 3. Results

### 3.1. Patient Demographics

**Figure 1:**
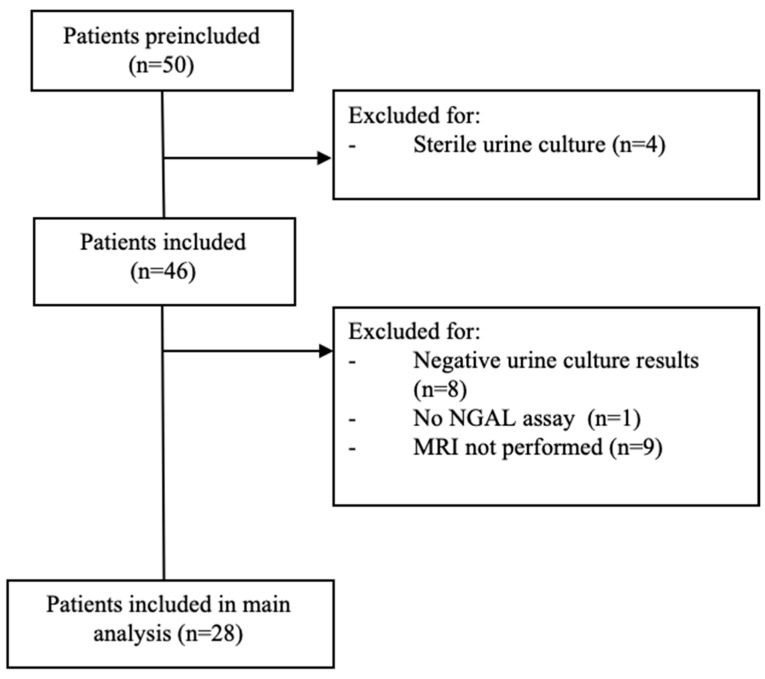
Study flow diagram. Abbreviations: MRI, magnetic resonance imaging; NGAL, neutrophil gelatinase-associated lipocalin.

Of the 50 children aged ≥ 4 years with a suspected UTI on admission during the study period, 28 met the inclusion criteria (Figure 1); 24 were from the APN group and 4 were from the lower UTI group. The median age was 9.6 years, and 78.8% of the patients were female (78.8%). The children in both groups had similar personal histories and sociodemographic, clinical, and paraclinical criteria (Table 1). Mean pNGAL, CRP, PCT, and WBC values were higher in the APN group, but the differences with the lower UTI group were not significant.

### 3.2. ROC Analyses

**Figure 2:**
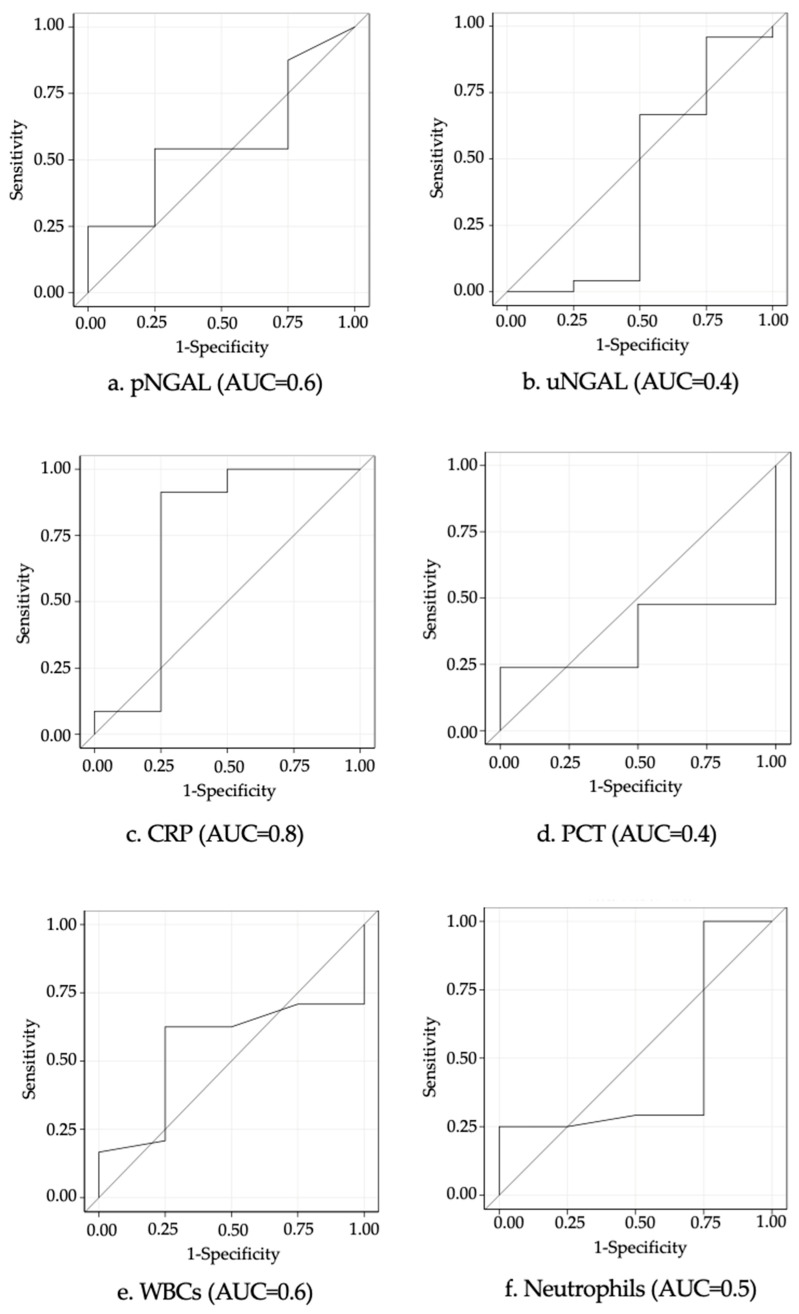
ROC analysis of pNGAL (**a**), uNGAL (**b**), CRP (**c**), PCT (**d**) levels, WBCs (**e**), and neutrophils (**f**) for predicting renal parenchymal involvement. Abbreviations: CRP, C-reactive protein; PCT, procalcitonin; pNGAL, plasma neutrophil gelatinase-associated lipocalin; uNGAL, urinary neutrophil gelatinase-associated lipocalin; WBCs, white blood cells.

The ROC curves for predicting renal injury (as visualized on renal–vesical MRI) based on biomarker levels in children with febrile UTIs are presented in Figure 2. CRP, pNGAL, and WBCs had the highest AUCs, with respective values of 0.8 (0.4–1.0), 0.6 (0.3–0.8), and 0.6 (0.3–0.8). The parameters indicating the diagnostic performance of the biomarkers are shown in Table 2. In the ROC analysis, the optimal cutoff for pNGAL was 141.0 ng/mL, which predicted renal parenchymal involvement with a sensitivity of 54.2% (34.2–74.1), a specificity of 75.0% (32.6–1.0), a positive predictive value (PPV) of 92.9% (79.4–1.0), and a negative predictive value (NPV) of 21.4% (0.0–42.3). The optimal cutoffs for the other biomarkers were 267.0 ng/mL for uNGAL, 2.3 mg/dL for CRP, 0.6 ng/mL for PCT, and 11.8/mm^3^ for WBCs.

### 3.3. Correlation Analysis

**Figure 3:**
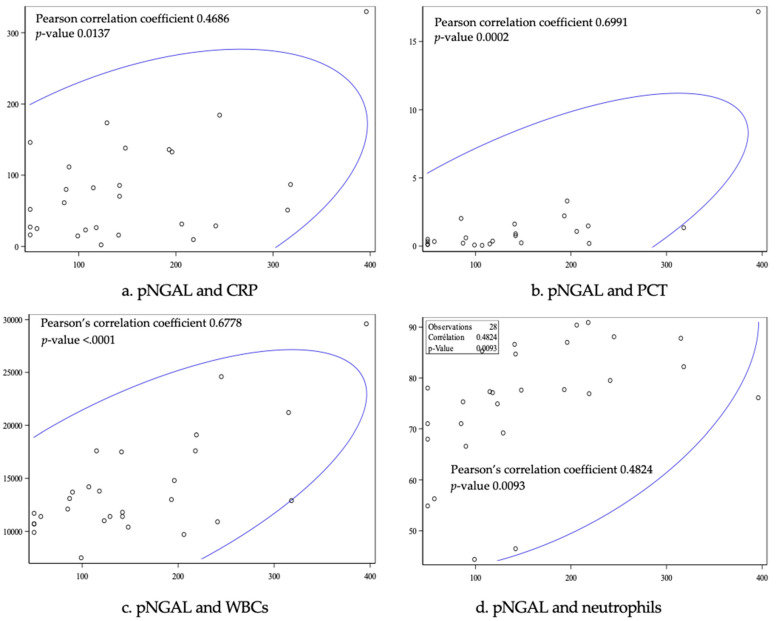
Correlation analysis between pNGAL and CRP (**a**), PCT (**b**), WBC (**c**), and neutrophils (**d**). Abbreviations: CRP, C-reactive protein; PCT, procalcitonin; pNGAL, plasma neutrophil gelatinase-associated lipocalin; WBCs, white blood cells.

The correlation analysis between pNGAL and the other biomarkers showed a Pearson’s correlation coefficient of 0.5 for CRP (*p* = 0.01), 0.7 for PCT (*p* = 0. 0001), 0.68 for WBC (*p* < 0.0001), and 0.5 for neutrophils (*p* = 0.009) (Figure 3). pNGAL was therefore positively, proportionally, and significantly correlated with PCT, WBCs, and neutrophils (*p* < 0.05).

### 3.4. Univariate Logistic Regression Analyses

None of the biomarkers were significant predictors of APN in the univariate analysis (Table 3). Multivariate logistic regression analysis was not performed due to the small sample size.

## 4. Discussion

In this study, we sought to analyze the ability of NGAL to accurately predict renal parenchymal involvement in children aged ≥ 4 years admitted with their first episode of febrile UTI. NGAL is an integral part of the innate immune system where it plays an important role in iron transport. The growth of Gram-negative bacteria, for example, is directly dependent on iron. Baseline NGAL levels are very low [29] but increase rapidly in response to cellular stress stimuli, such as ischemia, cytotoxins, and sepsis [30]. Patients with renal damage [15,31] or UTIs will thus experience a rapid rise in NGAL. Most febrile UTIs in children are caused by Gram-negative bacteria, and the diagnostic utility of NGAL in children with febrile lower UTIs or APN compared with control groups has already been demonstrated [17,32,33,34]. It is not clear, however, if NGAL can discriminate between upper and lower UTIs [22,23].

In our study, children with APN had higher pNGAL levels than those with febrile lower UTI, but the difference was not significant (Table 1). Our results suggest that pNGAL (AUC, 0.6) and uNGAL (AUC, 0.4) are not effective markers of APN. In the ROC analyses, the optimal pNGAL cutoff for predicting APN was 141.0 ng/mL, which is consistent with levels reported in other studies. In a study comparing 53 children with APN and 70 with lower UTIs (using DMSA scintigraphy as the gold standard), Sim et al. [33] reported an optimal pNGAL cutoff of 102.5 ng/mL. The corresponding cutoff reported by Kim et al. [32] in a retrospective study of 59 children with APN and 79 with lower UTIs was 117 ng/mL. The AUC for pNGAL in our study was lower than the values reported by Sim et al. (0.86) [33] and Kim et al. (0.89) [32]. The PPV was high, at 92.9%, but the other performance parameters were all lower (sensitivity, 54.2%; specificity, 75.0%; NPV, 21.4%). The studies by Sim et al. [33] and Kim et al. [32] differed from ours in terms of the type of assay used (whole blood immunoassay [Triage NGAL test]) and the age of the patients (median age of 4 years [33] and 70.3% of patients aged < 1 year old [32]).

In the two previously cited studies [32,33], the AUC for pNGAL was the highest of all the AUC values analyzed (CRP, WBC, and creatinine in the study by Sim et al. [33] and CRP, PCT, and WBC in that of Kim et al. [32]). It was very closely followed by the AUC for CRP, which, at 0.8, was the highest value in our study. At the optimal cutoff of 2.3 mg/dL, it had a sensitivity of 87.5%, a specificity of 75.0%, a PPV of 95.5%, and an NPV of 50.0%. These values are very similar to those reported by Kim et al. [32] for an almost identical cutoff of 2.8 mg/dL. CRP is a traditional marker of infection and is often used to diagnose febrile UTIs and evaluate treatment effectiveness. Based on our findings and those of previous studies [35,36], however, it does not appear to be a reliable marker for the diagnosis of APN due to its low specificity.

According to some studies, PCT is a more sensitive and specific marker than CRP for detecting APN [37], and levels > 0.5 ng/mL may even be predictive of renal scarring [38,39]. The AUC for PCT in our study, 0.4, was unexpectedly low. The optimal cutoff for predicting APN was 0.6 ng/mL (sensitivity, 47.6%; specificity, 50.0%). In the study by Kim et al. [32], the AUC for PCT was also lower than that observed for pNGAL and CRP, but, at 0.9, it was considerably higher than in our study. Kim et al. [32] also reported a lower cutoff (0.17 ng/mL). PCT thus does not appear to be a reliable marker of kidney damage in febrile UTIs in children.

uNGAL had a low AUC (0.42) in our study. Children with lower UTIs had a higher mean value than those with APN (971.0 vs. 524.5 ng/mL), but this may partly be due to the small size of the lower UTI group (just four patients). Unexpectedly, one of the patients in this group had an extremely high uNGAL value (3463 ng/mL), despite normal MRI findings and low levels of the other inflammatory biomarkers (pNGAL, 50 ng/mL, CRP, 1.6 mg/dL, PCT < 0.5 ng/mL). Several studies have shown uNGAL to be an effective diagnostic marker in children with febrile lower UTIs and APN in comparison with control groups [17,18,19,20,21,40,41]. uNGAL levels were also high in our two groups of patients. Contrasting findings on the ability of uNGAL to distinguish between upper and lower UTIs, however, have been reported. Ghasemi and al. [23] showed that uNGAL was a specific, but not sensitive, marker of renal damage in children with APN. Urbschat and al. [24], in turn, showed that adults with lower UTIs and APN had significantly higher uNGAL levels than controls, but the differences between the groups were insufficient to distinguish one infection from the other.

Although CRP was the strongest performer in our study, none of the biomarkers analyzed were able to predict renal damage in children with febrile UTIs. The lower values observed for pNGAL compared with the literature suggest that this biomarker is not an effective diagnostic tool. However, given its excellent PPV (92.9%), high pNGAL levels might be able to predict parenchymal damage with considerable accuracy. Further investigations with larger patient groups are required, and if its predictive performance is confirmed, pNGAL, which could be measured quickly in routine practice, could help clinicians determine the need for probabilistic antibiotic therapy while awaiting urine culture results. It could also help them choose between oral and intravenous antibiotic therapy. Given the low NPV, however, a pNGAL level below the cutoff of 141.0 ng/mL would unfortunately not exclude the presence of kidney damage. Nonetheless, in the correlation analysis, pNGAL correlated positively, proportionally, and significantly (*p* < 0.05) with all the other blood inflammatory biomarkers (CRP, PCT, WBC, neutrophils), suggesting that an approach combining all the markers could increase the probability of identifying renal lesions. Sim et al. [33] also found significant correlations between pNGAL and CRP and WBC.

Our study has some limitations. First, we recruited relatively few children. Recruitment was slow: on the one hand, because of the COVID-19 pandemic, and on the other hand, because APN most often affects very young children (our target population comprised children aged ≥ 4 years presenting with their first febrile UTI). Second, the reference standard used, MRI, may have missed some cases of APN that might have been detected by DMSA [25]. This could explain why there were no significant differences in characteristics between the groups. Our use of MRI probably also explains the small sample size, as an age cutoff of 4 years and older was chosen to avoid the need for sedation. Third, the children were recruited from just one hospital. We were unable to include patients from nearby hospitals (in Antibes and Grasse), as the study protocol required confirmation of renal lesions by pediatric radiologists, which was only available in Nice. Our results need validation in a larger prospective multicenter study comprising children of different ages. Future studies should incorporate the measurement of urinary creatinine to compensate for the variation in urine flow [19,40]. Despite the above limitations, our study has some strengths. First, it is one of few studies to analyze NGAL levels in children with UTIs aged ≥ 4 years, and second, we used a new automated NGAL quantification method, which would be easy to implement in everyday practice.

## 5. Conclusions

Parenchymal involvement in UTIs must be detected promptly and accurately to prevent renal scarring. In this prospective, single-center study, pNGAL and uNGAL were not found to be effective early predictors of renal parenchymal involvement in children with febrile UTIs. Nonetheless, pNGAL had an excellent PPV and could be a useful adjunct in the routine diagnosis of renal lesions. Caution, however, should be exercised when interpreting the findings of this pilot study given its small sample size. Larger studies are needed to clarify the role of NGAL in the diagnosis and treatment of febrile UTIs in children.

## Figures and Tables

**Table 1:** Study population characteristics.

		Total *n* = 28	Lower UTI *n* = 4	APN ^a^ *n* = 24	* *p*
**Demographics**	Age (years)	9.6 (4.6)	10.5 (4.7)	9.5 (4.7)	0.4739
	Male	21.4	25.0	20.8	1.0
	Female	78.8	75.0	79.2	1.0
**Personal history**	Weight loss	11.1	0.0	13.0	1.0
	Asthenia	53.9	50.0	54.6	1.0
	Eating difficulties ^b^	33.3	25.0	34.8	1.0
	Intolerable fever ^c^	54.2	25.0	60.0	0.3002
	Fever duration (days) ^d^	2.4 (3.2)	0.75 (0.25)	2.7 (3.4)	0.1142
	Fever peak	39.3 (0.7)	38.9 (0.5)	39.4 (0.7)	0.2242
**Physical Exam**	Asthenia	57.1	50.0	58.3	1.0
	Intolerable fever ^c^	17.9	0.0	20.8	1.0
	Pain requiring analgesics (>7 years old)	94.4	100.0	93.3	1.0
	Pain requiring analgesics (≤7 years old)	30.0	100.0	22.2	0.3000
**Dipstick**	Leukocyturia ^e^	61.6	50.0	63.6	0.6254
	Hematuria ^f^	44.0	50.0	42.9	1.0
	Nitrites	62.5	100.0	55.0	0.2589
**Biology**	WBCs (/mm^3^)	14,046 (4870)	12,925 (3130)	14,233 (5129)	0.7947
	Neutrophils (%)	75.1 (12.3)	76.5 (9.9)	74.8 (12.9)	0.7948
	CRP (mg/dL)	79.3 (73.1)	50.4 (82.3)	84.4 (72.2)	0.1367
	PCT (ng/mL)	1.5 (3.5)	0.99 (0.7)	1.6 (3.7)	0.5546
	Uremia (mmol/L)	4.0 (1.5)	4.13 (1.14)	4.0 (1.6)	0.7699
	Creatinine (mcmol/L)	48.6 (14.6)	43.5 (15.8)	49.5 (14.6)	0.3656
	pNGAL (ng/mL)	154.6 (89.8)	130.0 (68.8)	158.8 (93.4)	0.6963
	uNGAL (ng/mL)	588.3 (992.1)	971.0 (1666.1)	524.5 (873.9)	0.6264
**Type of antibiotherapy**	Intravenous	89.3	100.0	87.5	1.0
	Oral	10.7	0.0	12.5	1.0
**Type of care**	Hospitalization ^g^	42.9	50.0	41.7	1.0
	Outpatient	57.1	50.0	58.3	1.0

* *p* < 0.05 (by comparing sample “lower UTI” vs. “APN”). ^a^ Parenchymal involvement on MRI was defined by high signal intensity areas on the diffusion sequences and low signal intensity areas on the apparent diffusion coefficient maps; ^b^ Eating difficulties are defined as food intake < 50% of daily ration; ^c^ At least one of the following signs: cyanosis and/or chills and/or mottling and/or convulsions; ^d^ Number of days of fever before the emergency visit; ^e^ Positive for a rate > 10/mm^3^ (i.e., >10,000/mL); ^f^ Positive for a rate > 10/mm^3^ (i.e., >10,000/mL); ^g^ In any department. Tests used: Fisher exact test, chi-square test, or Wilcoxon rank sum test. Abbreviations: APN, acute pyelonephritis; CRP, C-reactive protein; PCT, procalcitonin; pNGAL, plasma neutrophil gelatinase-associated lipocalin; uNGAL, urinary neutrophil gelatinase-associated lipocalin; UTI, urinary tract infection; WBCs, white blood cells; MRI, magnetic resonance imaging.

**Table 2:** Predictive value of biomarkers for predicting renal injury using the ROC curve.

Biomarkers	AUC (95% CI)	Cutoff	Sensitivity (95% CI)	Specificity (95% CI)	PPV (95% CI)	NPV (95% CI)
**pNGAL** (ng/mL)	0.6 (0.3–0.8)	141.0	54.2 (34.2–74.1)	75.0 (35.6–1.0)	92.9 (79.4–1.0)	21.4 (0.0–42.9)
**uNGAL** (ng/mL)	0.4 (0.0–0.8)	267.0	37.5 (18.1–56.9)	50.0 (1.0–99.0)	81.8 (59.0–100.0)	11.8 (0.0–27.1)
**CRP** (mg/dL)	0.8 (0.4–1.0)	2.3	87.5 (74.3–1.0)	75.0 (32.6–1.0)	95.5.5 (86.8–1.0)	50.0 (10.0–90.0)
**PCT** (ng/mL)	0.4 (0.1–0.6)	0.6	47.6 (26.3–69.0)	50.0 (0.0–1.0)	90.9 (73.9–1.0)	8.3 (0.0–24.0)
**WBCs** (/mm^3^)	0.6 (0.3–0.8)	11.8	62.5 (43.1–81.9)	75.0 (32.6–1.0)	93.8 (0.8–1.0)	25.0 (0.5–49.5)
**Neutrophils** (%)	0.5 (0.1–0.8)	68	0.8 (0.6–1.0)	0.0 (0.0–0.0)	0.83 (0.7–0.9)	0.0 (0.0–0.0)

Abbreviations: AUC, area under the curve; CRP, C-reactive protein; PCT, procalcitonin; pNGAL, plasma neutrophil gelatinase-associated lipocalin; NPV, negative predictive value; PPV, positive predictive value; uNGAL, urinary neutrophil gelatinase-associated lipocalin; WBCs, white blood cells.

**Table 3:** Univariable logistic regression analysis for the prediction of renal parenchymal involvement.

		Crude Odds Ratio	*p*
**Biology**	**WBCs** (1000/mm^3^)	1.07 (0.82–1.41)	*p* =0.6173
	**Neutrophils** (%)	0.99 (0.90–1.08)	*p* =0.7959
	**CRP** (mg/dL)	1.01 (0.99–1.03)	*p* = 0.3915
	**PCT** (ng/mL)	1.08 (0.54–2.17)	*p* = 0.8223
	**pNGAL** (ng/mL)	1.00 (0.99–1.02)	*p* = 0.5508
	**uNGAL** (ng/mL)	1.0 (0.99–1.00)	*p* = 0.4146

Abbreviations: CRP, C-reactive protein; PCT, procalcitonin; pNGAL, plasma neutrophil gelatinase-associated lipocalin; uNGAL, urinary neutrophil gelatinase-associated lipocalin; WBCs, white blood cells.

## Data Availability

The data presented in this study are available on request from the corresponding author. The data are not publicly available due to privacy and ethical restrictions.
